# “We think globally”: the rise of Paraguay’s Tabacalera del Este as a threat to global tobacco control

**DOI:** 10.1186/s12992-018-0412-3

**Published:** 2018-11-19

**Authors:** Benoît Gomis, Kelley Lee, Natalia Carrillo Botero, Philip Shepherd, Roberto Magno Iglesias

**Affiliations:** 1 Faculty of Health Sciences, Simon Fraser University, Blusson Hall, 8888 University Drive, Burnaby, BC V5A 1S6 Canada; 2 Department of Management & International Business, College of Business, Florida International University, Modesto A. Maidique Campus, 11200 S.W. 8th St, MANGO 425, Miami, FL 33199 USA; 3 Center of Studies of Integration and Development, Rua Jardim Botanico 635/906, Rio de Janeiro, RJ 22470-050 Brazil

**Keywords:** Paraguay, Tobacco industry, Tabacalera del Este, Tabesa, Illicit tobacco trade, Tobacco control, Latin America

## Abstract

**Background:**

Leading transnational tobacco companies (TTCs) began to expand their operations in Latin America in the 1960s. This included legally exporting their cigarettes to Paraguay during the 1960s which, in turn, were illegally re-exported to Argentina and Brazil. By the 1990s, competition between BAT and PMI for this lucrative illicit market, focusing on low-priced brands, prompted manufacturing in Paraguay. Paraguayan manufacturing rapidly grew after the introduction of a new cigarette export tax in Brazil in 1999.

**Methods:**

We systematically searched Truth Tobacco Industry Documents (TTID) to understand the activities and strategies of leading TTCs in Paraguay and subregion over time. We applied the analytical framework of Lee and Eckhardt (2017) to understand Tabesa’s global business strategy. We searched the websites of TTCs and Tabesa for activities since the mid 2000s to understand how the companies publicly describe these strategies. We used the United Nations Commodity Trade Statistics Database (UN Comtrade) as an independent source to crosscheck statements by Tabesa executives about export markets. We contextualized and triangulated our findings with 42 key informant interviews.

**Results:**

Tabesa became the largest cigarette manufacturer in Paraguay, and one of the largest companies in the country, through complicity in the illicit trade. Enabled by market conditions created by leading TTCs, and a permissive regulatory environment in Paraguay, evidence suggests Tabesa had become a major source of illicit cigarettes across Latin America and beyond by the late 2000s. Although Brazil continues to account for the bulk of Tabesa’s revenues, findings suggest that the company is aspiring to compete with TTCs in markets worldwide through legal and illegal sales.

**Conclusion:**

There is a need for fuller understanding of the risks to global tobacco control from local companies aspiring to compete with TTCs. The rise of Tabesa is part of the changing nature of the illicit trade in tobacco products which must be taken into account in implementing the Framework Convention on Tobacco Control (FCTC) and its Protocol to Eliminate Illicit Trade in Tobacco Products. Potential conflicts of interest concerning Tabesa illustrate the importance of FCTC Article 5.3 on industry interference. There is also an urgent need to address the lack of independent and rigorous data on the illicit tobacco trade in the region.

## Resumen

**Introducción:**

las principales empresas tabacaleras transnacionales (TTCs por sus siglas en inglés) comenzaron la expansión de sus operaciones en América Latina durante la década de 1960. Esto incluyó la exportación legal de cigarrillos a Paraguay durante los años sesenta, para ser posteriormente reexportados ilegalmente a Brasil y Argentina. En la década de los noventa, la competencia entre BAT y PMI por este lucrativo mercado ilícito, centrado en marcas de bajo precio, incentivó la producción en Paraguay. La producción paraguaya creció rápidamente después de la introducción de un nuevo impuesto a las exportaciones de cigarrillos en 1999 en Brasil.

**Métodos:**

Efectuamos búsquedas sistemáticas en los documentos de la industria tabaquera (Truth Tobacco Industry Documents (TTID)) para entender las actividades y estrategias de las principales TTCs en Paraguay y en la subregión a través del tiempo. Utilizamos el marco analítico de Lee y Eckhardt (2017) para analizar la estrategia comercial global de Tabesa. Examinamos las páginas web de las TTCs y de Tabesa desde mediados de la década de 2000 para comprender cómo estas empresas describen públicamente sus estrategias. Utilizamos la base de datos COMTRADE sobre estadísticas de comercio internacional de mercaderías como una fuente independiente para cotejar con declaraciones de los ejecutivos de Tabesa sobre los mercados de exportación. Contextualizamos y triangulamos nuestros hallazgos con 42 entrevistas con informantes clave.

**Resultados:**

Tabesa se convirtió en el productor de cigarrillos más grande de Paraguay y en una de las compañías más grandes del país gracias, en parte, a su complicidad con el comercio ilícito. Facilitado por las condiciones del mercado creadas por las principales TTCs, así como por un entorno regulatorio permisivo en Paraguay, la evidencia sugiere que, para fines de los años 2000, Tabesa se había convertido en un importante proveedor de cigarrillos ilícitos en América Latina y fuera de la región. Aunque el comercio con Brasil sigue representando la mayor parte de los ingresos de Tabesa, los hallazgos sugieren que la compañía aspira a competir con las TTCs en los mercados mundiales a través de ventas tanto legales como ilegales.

**Conclusión:**

es necesario lograr una comprensión más completa de los riesgos que representan las compañías locales que aspiran a competir con las TTCs para el control mundial del tabaquismo. El ascenso de Tabesa es parte de la naturaleza cambiante del comercio ilícito de productos de tabaco, y debe tenerse en cuenta en la aplicación del Convenio Marco de la OMS para el Control del Tabaco (CMCT OMS) y su Protocolo para la eliminación del comercio ilícito de productos de tabaco. Posibles conflictos de interés relativos a Tabesa ilustran la importancia del Artículo 5.3 del CMCT sobre la interferencia de la industria. También es urgente abordar la falta de datos independientes y rigurosos sobre el comercio ilícito de tabaco en la región.

## Introduction

Since the 1990s, there has been an accelerated restructuring of production and consumption within the tobacco industry worldwide, and further concentration of ownership in the hands of a small number of leading transnational tobacco companies (TTCs), as part of processes of tobacco industry globalization [1]. By 2016, 79.6% of the world cigarette market outside China was controlled by six TTCs [2]. Public health research to date has thus focused on the role of these companies in contributing to the growing rates of tobacco use in many emerging markets, and the corresponding rise in tobacco-related disease and death during this period [3]. Far less, however, is known about the business strategies of tobacco companies that have remained independent of leading TTCs [2], amid tobacco industry globalization, and the implications for global tobacco control [4, 5].

This paper examines one of the few remaining independent tobacco companies in Latin America, Paraguay’s Tabacalera del Este (known as Tabesa). Tabesa is owned by businessman Horacio Cartes, Paraguay's elected president between 2013 and 2018. When the company was established in 1994, the Paraguayan company La Vencedora, and leading TTCs British American Tobacco (BAT) and Philip Morris International (PMI) held 41%, 32%, and 22% of the domestic market share respectively [6]. As well as being the largest cigarette manufacturer in the country, today Tabesa is one of only two Latin American tobacco companies exporting to the US market. Building on our accompanying analysis of the tobacco industry in Paraguay from the 1960s to the early 2000s, focused on how the country was used as a major transit hub for illicit re-export of TTC brands to Brazil and Argentina [7], the purpose of this paper is to analyse the regional and global business strategies of Tabesa since the mid 1990s. We explain how Tabesa has grown to dominate the domestic market, then became a major regional company, and more recently expanded into an aspiring TTC, by analysing the key internal and external factors behind Tabesa’s growth. This includes the central role played in this growth by the illicit tobacco trade which the World Health Organization (WHO) Framework Convention on Tobacco Control (FCTC) Protocol to Eliminate Illicit Trade in Tobacco Products defines as “any practice or conduct prohibited by law and which relates to production, shipment, receipt, possession, distribution, sale or purchase including any practice or conduct intended to facilitate such activity” (Article 1). The previous complicity of leading TTCs in the illicit tobacco trade, estimated to account for 9–12% of cigarette consumption worldwide [8, 9], has been well documented [10–18]. Far less is known about the role of non TTCs in the illicit trade. While there have been longstanding media reports that Tabesa is one of the primary sources of smuggled cigarettes in Latin America [19–22], this paper provides the first detailed and scholarly account of this alleged activity, and how it fits with the company’s business strategy within the context of tobacco industry globalization. We conclude by considering the implications of Tabesa’s growth for implementing the FCTC and Protocol.

## Background

Latin America became an important region for TTC expansion during the 1960s. Growing concerns about the accumulating evidence of the adverse health effects of tobacco use led to the decline in smoking prevalence in so-called traditional markets such as the US [23]. To compensate for anticipated declining sales in these markets, TTCs turned their attention to emerging markets.

In many Latin American countries, market access to many industrial sectors was tightly controlled by import substitution policies. Many sectors were nationally-owned and thus protected – including restrictions on foreign direct investment (FDI) and consumer goods imports - limiting the scope for foreign companies, particularly in the consumer-goods sector, to operate in these markets [24–26]. In our accompanying paper [7], we argue that Paraguay became an important destination for substantial volumes of legally exported TTC-manufactured cigarettes which were then illegally re-exported to Brazil and Argentina. Paraguay’s role, as “a staging point for re-exports” [27], enabled TTCs to establish a strong regional presence and demand for their brands. Specifically, we identify four phases in the illicit tobacco trade involving Paraguay: 1) as a transit hub for smuggling BAT and PMI cigarettes from the U.S. into Argentina and Brazil (from the 1960s to the mid-1970s); 2) BAT and PMI competing in the north-east of Argentina (1989-1994); 3) BAT and PMI competing in southern and south-eastern Brazil (mid to late 1990s); and 4) growth in the illicit trade of Paraguayan manufactured cigarettes (from the mid-1990s). In short, TTCs had shifted from supplying premium brands to becoming complicit in the supply of lower-priced, untaxed cigarettes in Brazil and Argentina via the illicit trade. The introduction of a 150% cigarette export tax by Brazil in 1999, to discourage the illegal re-export of Brazilian manufactured cigarettes back into the country, effectively ended the use of Paraguay by TTCs as a transit hub. The combined creation of a substantial market in Brazil and Argentina for low-priced cigarettes, and the Brazilian export tax, led to a boom in domestic cigarette production in Paraguay [7].

It is in this context that we seek to understand the rise of Tabesa from the early 2000s, including its role in the illicit tobacco trade. Cigarette manufacturer Tabesa is part of the Grupo Cartes conglomerate built by siblings Horacio and Sarah Cartes. Tabesa operates alongside Tabacos del Paraguay (a distribution company created in 1997 and renamed Palermo in August 2016) [28], Compañía Agrotabacalera del Paraguay (a leaf cultivator which has supplied Tabesa since 2002), Valla Global Ventures (supporter of Tabesa’s international expansion founded in 2002), Tabacos USA (active since Tabesa’s expansion to the US in 2008), and Cigar Trading and Habacorp (a cigar business founded in 1999). In 2015, Tabesa was the country’s largest corporate taxpayer (around US$45 million) [29, 30].

Ownership and structure of the tobacco industry in Paraguay is unusual for Latin America. BAT and PMI have acquired most of the region’s local companies through mergers and acquisitions (M&As). By 2017 BAT and PMI controlled 83% of the region’s cigarette market (43.2% and 39.8% respectively) [31]. In contrast, Paraguay is one of only three countries, along with Uruguay (Cía Industrial de Tabacos Monte Paz) [32] and Bolivia (Cía Industrial de Tabacos CITSA and Bis Overseas Bolivia SRL) [33], to still have locally-owned companies with a larger market share than TTCs. Tabesa accounted for half of Paraguay’s tobacco products market in 2015 [34–37]. How and why this has come to be the case, and what this means for global tobacco control, has been little studied to date.

## Methods

To determine what is currently known about the tobacco industry in Paraguay, we began by reviewing the existing English, Spanish and Portuguese language scholarly literature published since 1990 using JSTOR and Google Scholar, with the keywords “Paraguay” combined with “Tabesa”, “Tabacalera del Este”, “tobacco”, “cigarettes”, and “smuggling” (and related terms). This search extended to the following subject areas: business, criminology, economics, geography, history, international relations, Latin American studies, political science, public health, and public policy and administration. A total of 154 relevant scholarly papers were identified. We then used the same keywords to search the Factiva and Lexis Nexis databases of media sources. A total of 467 relevant media articles were identified, dating between 1990 and 2016. We also reviewed industry and business news sources such as *Tobacco Reporter* (www.tobaccoreporter.com), KPMG (www.kpmg.com), Euromonitor International (www.euromonitor.com), Panjiva (www.panjiva.com), and MarketResearch.com.

To understand the strategies of leading TTCs in Paraguay, we systematically searched internal industry documents available from the Truth Tobacco Industry Document (TTID) collection using English and Spanish language keywords. We initially used the keywords “Paraguay” combined using Boolean terms with “Tabesa”, “Tabacalera del Este,” “Boqueron,” “La Vencedora,” “Tabacos USA,” “Tabacos del Paraguay” and “Palermo”, to identify documents related to the domestic industry. We then combined “Paraguay” with “DNP” (duty not paid), “transit” and other known euphemisms for the illicit tobacco trade [14, 38]. We used the snowball technique to generate additional search terms such as names of individuals, brands and projects [39]. A total of 1239 documents were identified and reviewed for relevance to the business strategies of Tabesa. Given that most TTID documents date from before the mid 2000s, we consulted other industry sources for more recent information on company strategies and activities. We systematically searched the company websites of Grupo Cartes (http://www.grupocartes.com.py/), Tabacalera del Este (http://www.tabesa.com.py), Tabacos del Paraguay (http://www.tabacosdelparaguay.com), Palermo (http://palermo.com.py), Tabacos USA (http://www.tabacosusa.com), BAT (www.bat.com.py/) and PMI (https://www.pmi.com).

It is important to note that accurate measurement of the illicit tobacco trade worldwide remains difficult given the nefarious nature of the activity, varying definitions, limited availability of official and independent data sources, and unreliability of methods [8, 40]. For example, estimates based solely on customs seizures can be misleading, reflecting increased law enforcement efforts rather than actual changes in illicit trade volumes [41–43]. In Latin America, we also found that industry-linked data sources are frequently uncritically cited by the media, researchers, and even government officials. However, as well as substantial evidence of TTC complicity in the illicit trade [10–18], there is evidence that TTCs have promoted over and/or underestimates of the scale of the illicit trade, and its causal factors, to serve industry interests and undermine tobacco control measures [44, 45]. In some contexts, the tobacco industry even provides training to customs and law enforcement officials on how to detect illicit tobacco products, notably counterfeits which compete with their products [46]. Overall, the lack of comprehensive, independent and reliable data on the illicit tobacco trade in Latin America over time has been a major challenge to analysing and addressing its causes.

To address this problem, we analysed the UN Commodity Trade Statistics Database (UN Comtrade) (http://comtrade.un.org) for independent data on the legal tobacco trade. This database “stores standardised official annual trade statistics reported by countries and reflecting international merchandise flows detailed by commodity and partner country with coverage reaching up to 99 percent of world merchandise trade” [47]. Using data on commodities ‘240220 - Cigarettes; containing tobacco’, we compared reported imports from Paraguay to reported exports to eleven markets worldwide. The markets were identified in statements by Tabesa employees in the Paraguayan media: Aruba, Bulgaria, Curaçao, Mongolia, the Netherlands, the Netherlands Antilles,^1^ Romania, Spain, Switzerland, UAE,^2^ and US from 2001 to 2016. We used the conversion rate applied in Iglesias et al. [48] to estimate the number of cigarette sticks from net weight (1 kg = 956.4 cigarettes). The difference between official exports and imports is assumed, in this analysis, consistent with Merriman [44], to be largely composed of illicit trade.

In applying this method, to estimate the illicit tobacco trade involving Paraguay, we acknowledge that “trade statistics - as any source of information - are not free of mistakes and omissions” [49]. According to the World Bank, there are four reasons for discrepancies between official exports and imports: (a) imports are recorded cif (cost insurance and freight) while exports are fob (free on board) which may differ by 10–20% in value; (b) data quality may vary by country; (c) imports are usually recorded with greater accuracy because they generate tariff revenues; and (d) the same good may be recorded in different categories by the exporter and importer [50]. Despite these caveats, Merriman notes that “persistent discrepancies between these amounts - discrepancies that cannot be explained by other factors - provide an estimate of the amount of wholesale smuggled tobacco” [51].

To contextualize and triangulate our findings, we conducted 42 key informant interviews with scholars, activists, consultants, law enforcement officials and government representatives from the region by telephone between November 2014 and February 2017, and during a visit to the region in June 2015. Key informants were initially identified through the literature review, and then the snowballing method was used to identify further interviewees. Given the subject matter of this research, only two interviewees consented to be on the record (although their contributions do not form part of the final paper). Two other interviewees agreed to be quoted anonymously. Where key informants provided consent to be quoted, compliant with recognized ethics guidelines [52, 53], and ethics approval from the Office of Research Ethics, Simon Fraser University, we cite key informants using generally worded attributions to maintain anonymity. The remaining key informants only consented to speak off the record. Information from these interviews provided useful background and was used to triangulate findings derived from other sources.

The above sources were compiled and triangulated, thematically and chronologically, to derive a narrative of Tabesa’s business strategy and activities. To organize this material, we applied the framework of Lee and Eckhardt [4] heuristically to structure our analysis of its global business strategies focused on three questions: (a) what are the main factors shaping a company’s global business strategy? (b) what are the specific strategies pursued to achieve globalisation? and (c) to what extent has the industry as a whole, or the specific company in question, globalised to date?

## Results

### “Replacing the market the multinationals abandoned”: Internal and external factors shaping Tabesa’s business strategy

Lee and Eckhardt argue that various internal or external factors may explain a tobacco firm’s pursuit of globalization. Internally, a firm may seek: (a) *natural resources*; (b) *markets*; (c) *improved efficiency*; or (d) *strategic assets* [4]. Applying this framework, internal industry documents suggest the motivation for Tabesa’s expansion beyond Paraguay was primarily as a market seeker, aiming “to expand the sale of goods and services beyond the domestic market” [4]. The small size of the domestic market in Paraguay suggests that it was not the driver of Tabesa’s growth. Indeed, adult (> 15 years) smoking prevalence in Paraguay has been relatively low, and declined from 24% in 1995 [54] to 14.5% in 2011–2016 [55, 56]. Retail sales of tobacco products in Paraguay shrank from US$149 million in 2002 to US$62 million in 2016 (in 2016 US$) [57]. In 2013, there were fewer than 570,000 adult daily smokers among Paraguay’s 7 million population [58, 59]. As described in our accompanying paper [7], from the early 2000s, Paraguayan tobacco manufacturing grew to fill a vacuum in the illicit supply chain to Brazil and Argentina left by leading TTCs [7]. Subsequently a downward trend in adult smoking prevalence in Brazil, from 35% in the late 1980s to 15% in 2008 [60], and Argentina, from 40% in 1999 [54] to 25% in 2016 [56], led Tabesa to seek markets beyond the subregion. Since the mid 2010s, Tabesa has sought “to boost exports to the Caribbean and expand sales to other markets in Asia and Europe” [61]. With company executives believing that “a good deal may come from anywhere in the world” [62], available data presented below suggests a rapid diversification by Tabesa of its markets beyond the subregion.

While internal factors influenced the markets that Tabesa and other Paraguayan tobacco companies targeted, evidence suggests that two external factors explain the emergence and growth of these companies: (a) *market conditions* (e.g. degree of openness, competition, preferential trade and investment agreements); and (b) *regulatory environment* (e.g. tobacco control measures, law enforcement, customs) [4]. First, documents suggest that leading TTCs created enabling market conditions in the subregion that channelled legal exports to the illicit re-export market [63]. Using Paraguay as “a staging point for re-exports” beginning in the 1960s [7], documents suggest BAT and PMI established a flourishing business model that integrated the legal and illegal trade. From 1989 onwards, the two companies then shifted their focus to lower-priced brands. The tripling of Paraguayan manufacturing during 1995–1998 was a direct response to this new market opportunity. A further doubling in production from 1999 to 2003, to then fill a drop in supply from leading TTCs, due to the Brazilian cigarette export tax of 1998, made Paraguayan companies direct competitors of TTCs [7]. Documents suggest Tabesa used the same distribution networks, previously used to illegally “re-export” TTC brands back into Brazil, for its own products [64, 65]. According to Tabesa’s Chief Executive Officer (CEO) José Ortiz, the company was merely “replacing the market the multinationals abandoned”, calling TTCs “the parents and grandparents of the creature” [translated by the authors] [66]. Ortiz described subsequent TTC accusations of illegal activity by Tabesa as “moral hypocrisy” given that “the multinationals in Brazil and Argentina are the ones who used Paraguay to export their products so that Brazilians and Argentines would come to Paraguay and buy our products” [translated by the authors] [66]. Similarly, Cartes stated,

I want [BAT and PMI] to show me what they said when they exported all their cigarettes back to Argentina [and Brazil]….The [Paraguayan] Ministry of Finance informed Brazil when Souza Cruz was exporting 140,000 cases per month here, much more than we are selling [today]. You need to see what they said when they were exporting. If there is a market today, they generated it. If they come looking for it today, it is because they generated it. [translated by the authors] [22].

A second external factor in Tabesa’s rapid growth was a permissive regulatory environment. The so-called “Triple Frontier” (shared by Paraguay, Argentina and Brazil) has long been an important transit zone for smuggling given the presence of multiple border entry points by air, land and water, along with weak border controls. A 1990 study of Paraguayan trade found that “contraband accounted for 58% of exports and 31% of imports” [67]. This included a substantial role in the illicit drug trade. Paraguay is among the largest cannabis producers in the Western Hemisphere, and is a transit hub for cocaine en route to Brazil, Argentina, West Africa, Europe and the Middle East [68]. With Tabesa’s factory located close to the Iguazú and Paraná Rivers, and the Friendship Bridge linking Ciudad del Este in Paraguay and Foz do Iguaçu in Brazil, the company has been well positioned geographically to transport its goods across this border area.

It is in this context, of a substantial and thriving illicit tobacco trade between Brazil and Paraguay, that in 1999 the Brazilian Ministry of Finance introduced a 150% tax on cigarette exports to Latin America, in an effort to stem their tax-free “re-export” back into the country via illicit channels [48]. As detailed in our accompanying paper, the new tax led to a sharp decline in Brazilian cigarette exports to Paraguay and, in turn, a significant increase in cigarette production in Paraguay for export [7]. Production rose in volume by a remarkable 2592% between 2000 and 2010 [69], much of which was reportedly exported to Brazil and other countries illegally [62, 70, 71]. Overall, documents suggest that the rapid expansion of Tabesa’s manufacturing capacity was to fill this void in the supplying of the illicit trade.

### “We think globally”: Mimicking selected business strategies of leading TTCs

Among the different ways that Tabesa could have pursued “various global markets” [translated by the authors] [72], our findings suggest three main strategies. First, Tabesa created new brands (e.g. *Rodeo*, *Eight*), manufacturing them domestically, and then exporting them via illicit channels [62]. When this business proved highly successful, and the company grew rapidly as a result, Tabesa began to “think globally” about product development [73]: “We developed our tobacco blends with international characteristics...national production was adapted to international requirements in order to compete on quality” [translated by the authors] [34]. Tabesa has also built “state-of-the-art modern factories” [70] to produce more cigarettes to this higher standard that could then compete for new markets abroad.

By the mid 2000s, therefore, Tabesa’s business strategy had changed. A higher quality and quantity of Tabesa brands by then were being produced, still largely for the illicit market, but now not only for the subregion, but for markets further afield. According to WHO, Paraguay had emerged as “a top producer of contraband tobacco” [9]. In 2006, Paraguayan companies produced 68 billion cigarettes which Ramos [74] estimates represented approximately 11% of the world’s total illicit trade. As this was more than twenty times the amount consumed domestically, law enforcement officials estimated that up to 90% of cigarettes manufactured in Paraguay (worth around US$1 billion) was being produced for smuggling to other countries [74]. Brazil remained a key destination. As one former law enforcement official described, it is the focus of “70% of Tabesa’s activity… Paraguayan factories like Tabesa take part in smuggling. They know the clients, they facilitate it. They create companies all over the place and the product then disappears” [70]. Beyond Brazil, there is evidence that Free Trade Zones (FTZs) have provided new opportunities to divert cigarettes to the illicit market given their limited regulation and customs presence [75]. For example, Tabesa’s sister company, Valla Global Ventures (VGV), is described on the Grupo Cartes company website as “a Society constituted under the laws of the British Virgin Islands” and “a Trading Company… dedicated to selling and exporting tobacco products to different world markets”, focusing on “commercialization and international intermediation for the sale of cigarettes” [translated by the authors] [76]. For instance, in 2009 and 2010, VGV imported amounts of cigarettes manufactured by Tabesa to the British Virgin Islands and the Corozal FTZ in Belize that far exceeded local levels of consumption [77, 78].

Alongside business strategies reminiscent of leading TTCs, Tabesa executives have adopted arguments previously used by TTCs to publicly defend themselves against accusations of complicity in the illicit trade. For example, recent media reports suggest criminal organizations such as Los Zetas and the Sinaloa cartel in Mexico, the Urabeños and the Revolutionary Armed Forces of Colombia (FARC), and the Red Command and the First Capital Command (PCC) in Brazil have been involved in the distribution of illicit Paraguayan cigarettes, using the trade to launder revenues from trafficking of cocaine, heroin and methamphetamines [79–81]. Similar to leading TTCs [82], Tabesa officials have claimed that, after legally selling to distributors, the company is not responsible for the onward sale of its products illegally. In a 2009 interview, Ortiz argued, “We have the function of supplying the market: If you have a butcher shop and someone likes your ribs, will you stop selling if you have the meat?... We have no way of knowing where our cigarettes are smoked, nor is it our problem” [translated by the authors] [66]. In a 2016 interview on cigarette smuggling in Colombia, Ortiz similarly argued that “the problem is theirs, they have a tax problem… [Tabesa] does not owe investigators any explanations about why cigarettes made by Tabesa were smuggled to Colombia” [translated by the authors] [83]. According to Cartes, the problem lies with customs officials in destination countries: “For me, contraband is a customs problem. We don’t do any of it; we have a clear conscience. It is the same as Nestlé. They are not responsible for who sells Nido milk here” [translated by the authors] [22].

A third way Tabesa has pursued its business strategy has been to establish a legal presence in the US market. In 1999 the company applied for trademark protection of its *Palermo* brand with the US Patent and Trademark Office (USPTO) [84]. Tabesa describes the granting of trademark protection in 2001 as coming after years of “intense negotiations”, and in 2007 “the prestigious certification…to sell cigarettes in this demanding market” [translated by the authors] [61]. Tabacos USA began selling *Palermo* in February 2008. The websites of Grupo Cartes, Tabesa, and Tabacos USA describe these developments in glowing terms:

One of Tabesa’s products conquered the US market, where all states had to approve its commercialization under a regime called the MSA [Master Settlement Agreement]. This modality is only for companies that qualify for strict procedures and demanding controls. The US states gave their approval for Tabesa to sell cigarettes in that demanding market. In all of Latin America, only two companies are part of MSA: Montepaz from Uruguay and Tabacalera del Este [translated by the authors] [34].

As stated on the Grupo Cartes website by Juan Carlos López Moreira, head of Tabacos del Paraguay at the time and a key player in the negotiations: “We worked hard. It was a long journey. I remember the day they gave us the certification very well. At 10.15 pm on December 27, 2007, on my birthday, the US attorney called me telling me that we had been awarded the MSA certification [translated by the authors]” [85]. The thoroughness of the certification process is described on the company website:

Months earlier, in Washington, prosecutors had subjected López Moreira [then CEO of Tabacos del Paraguay] and Barriocanal [then a senior executive in the Cartes Group and CEO of Bebidas del Paraguay] to all sorts of questions about Horacio Cartes’ companies and their trajectory for 8 hours. They had to back up everything with documents and even addressed queries about media publications against them. Even then, they left the files with the prosecutors for them to investigate everything. [translated by the authors] [61].

**Fig. 1:**
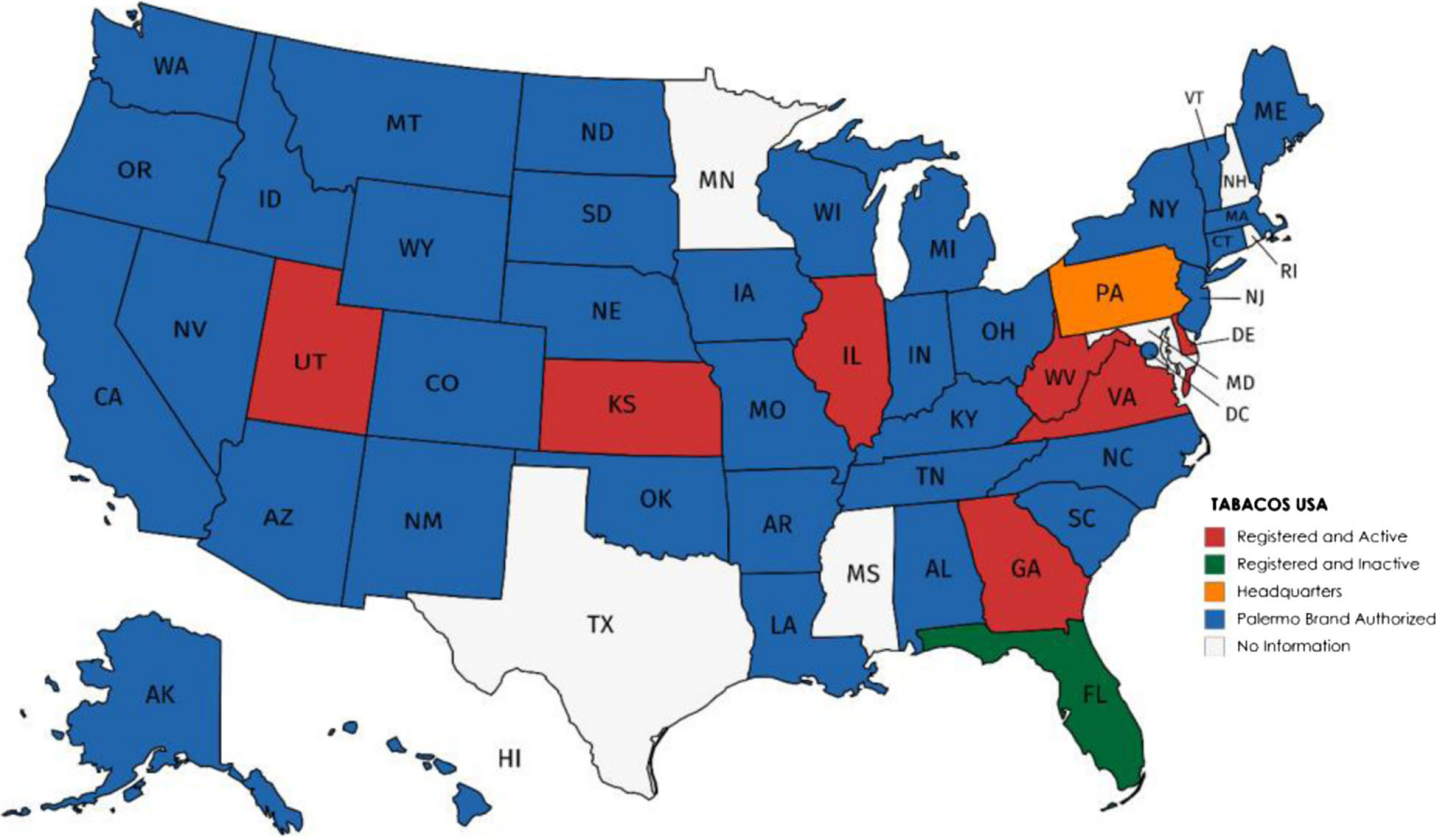
Tabesa’s market presence by US states. Source: Compiled from state-managed business registration databases for Tabacos USA, Tabesa and Tabacalera del Este [accessed in February 2017]

However, evidence suggests this expansion into the US market has not been to generate profits through American sales. While Grupo Cartes has expressed “satisfaction to know that sales [of *Palermo* in the US] are superior to those in Paraguay” [translated by the authors] [61], to date, the US market for *Palermo* has been modest. In a 2012 media interview, Moreira indicated that Tabesa was just “breaking even” [translated by the authors] in the US, and argued that this left little margin for alleged money laundering [86]. Data on shipments from Tabesa between 28 April 2016 and 23 December 2016 show exports of 38,913 kg of cigarettes to the US (or 37.2 million cigarettes using Iglesias et al. methodology [48]), approximately one-thirteenth of its exports to Aruba (524,179 kg or 501.3 million cigarettes) [87]. Annual revenue of Tabacos USA in 2016 was a relatively modest US$1.8 million [88]. Although Tabesa’s 2012 report to the US Patent and Trademark Office indicates that its *Palermo* “is presently being sold in the Commonwealth of Pennsylvania and in some 25 other states through its importer and distributor, Tabacos USA” [89], a comprehensive business search shows that the company is registered and active in only seven US states (Fig. 1). This suggests Tabesa has been less financially successful in the US market than the company claims.

Evidence suggests that, instead of sales, permission to export to the US market has been more important as a source of legitimacy for the company and its executives, and used as a defence against allegations of cigarette smuggling, money laundering and drug trafficking [90, 91]. As stated by Cartes in a media interview on the US government’s investigation of his business operations,

When there are two companies in all of Latin America that can export to the US, Montepaz from Uruguay and Tabacalera del Este…I can assure you that the controls are extraordinarily strict….It was a process of more than 3 years, because there are 51 [sic] states that have to authorize you….If we are going to be falsifying exports to the US with the controls they have in place… [translated by the authors] [22].

During the 2013 presidential elections, Senator Julio Velazquez of Paraguay’s Colorado Party similarly evoked the certification process to defend Cartes in a media interview: “When it comes to drug trafficking, Horacio has made it clear what his position is…There’s no concrete allegation against him. Horacio has investments in the US. Do you think the Americans would allow a narco to bring money into their country?” [92].

### How globalized is Tabesa to date?

**Fig. 2:**
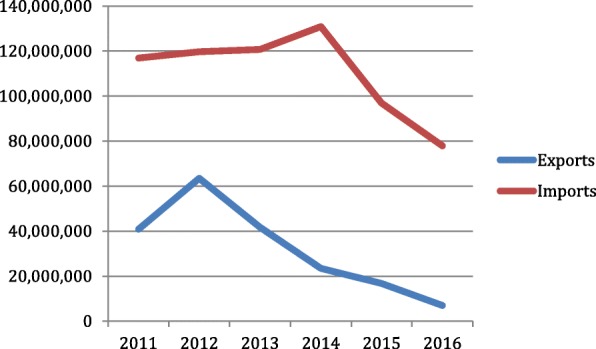
Tabesa total exports and imports, 2011–2016. Source: Compiled from Departamento Estadistica, Adminstración Sistems SOFIA, Dirección Nacional de Aduanas [accessed in June 2017]

**Fig. 3:**
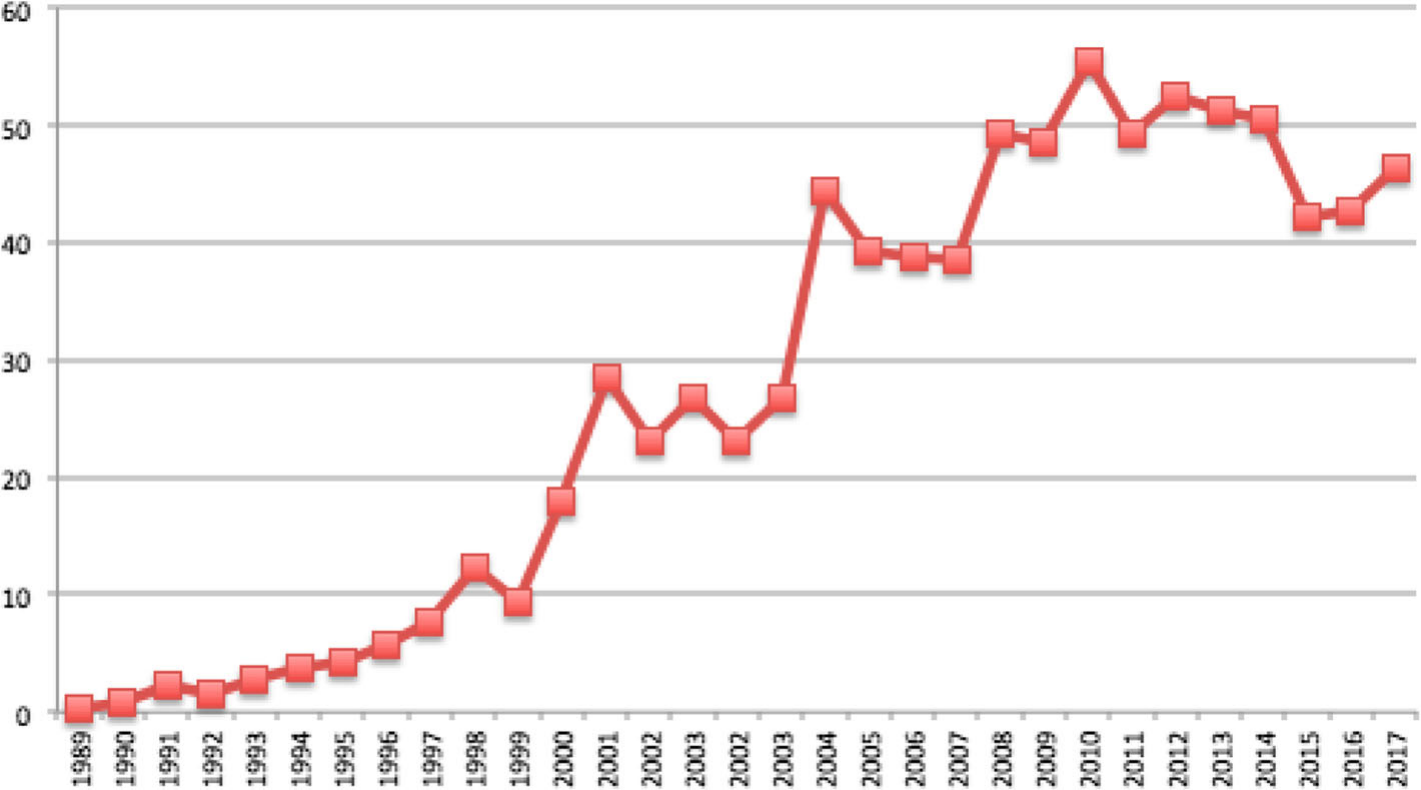
The continued growth of Paraguayan cigarette production (in billion cigarette sticks). Source: Estimated from UN Comtrade data and methodology used in Iglesias et al. [48]

Officially registered exports by Tabesa have decreased by 89% since 2012 and its registered imports of inputs for cigarette manufacturing have declined by 40.5% since 2014 [93] (Fig. 2). At first glance, this seems to indicate that Tabesa’s expansion has stalled. However, close analysis of available additional data suggests otherwise. Tabesa has remained Paraguay’s largest corporate taxpayer, cigarette manufacturer and leader in domestic sales, outperforming BAT and PMI [29, 37, 30]. Tabesa’s *Kentucky* is the bestselling brand in the country [94]. In 2015, the company imported approximately 2.8 million kilograms of acetate tow, 1.3 million kilograms of cigarette paper, 320,000 kg of filter paper, 875,000 kg of tipping paper, and 247,000 kg of triacetin, enough to produce between 25 and 36 billion sticks (Table 1). Given that Paraguay’s domestic consumption is around 4 billion sticks, and official legal exports are 2.1 billion cigarettes [95], this suggests 19–30 billion cigarettes manufactured by Tabesa may be entering the illicit market annually. Additional data compiled by the authors, using the estimation methodology based on tobacco leaf imports described in our accompanying paper [7], suggests that Paraguayan cigarette manufacturing continues to grow (Fig. 3). UN Comtrade data, showing that Paraguayan imports of cigarette paper (HS 4813) and cellulose acetate (HS 391211 and 391,212) - two key cigarette inputs – respectively increased by 40% and 52% between 2012 and 2017, suggests the same trend.

**Table 1:** Tabesa imports of key cigarette components (2015). Source: Compiled from data provided courtesy of BITQ Consulting Services Limited, 2015–2016

Cigarette components	Tabesa imports in kgs	Equivalent in bn cigarette sticks
Acetate tow	2,759,406.90	24.88
Cigarette paper	1,322,541.10	35.69
Filter paper	319,549.40	25.30
Tipping paper	847,793.00	36.24
Triacetin	246,800.00	28.19

There is also additional evidence that large volumes of cigarettes continue to be exported to Brazil illegally from Paraguay. According to a law enforcement official, Brazil accounts for 70% of Tabesa’s profit [70]. Despite Tabesa having no officially reported cigarette exports to Brazil, Brazilian customs authorities seized 341.1 million packs of cigarettes coming from Paraguay between 2010 and 2013 [96]. Brazil’s Department of Federal Revenue reported that, of the 70 Paraguayan brands smuggled into Brazil, five were manufactured by Tabesa and accounted for 49% of total seizures [97]. Similarly, Iglesias et al. found that “the total proportion of illicit cigarette consumption and the total amount of yearly illicit manufactured consumption [in Brazil] increased from 16.6% to 31.1% and from 13.0 to 24.3 billion of units, respectively” between 2008 and 2013 [98].

Other sources report that Tabesa brands have been illegally exported to other Latin American countries. Ramos reported in 2009 that *Rodeo* and *Eight* were the most frequently confiscated in Argentina, with around 45% of cigarettes smuggled into the country coming from Tabesa [74]. In November 2014, the Colombian newspaper *El Espectador* published a detailed account of the alleged contraband routes for tobacco in South America, highlighting Paraguay as the region’s key production hub and Tabesa as its main manufacturer [99]. In Colombia, Tabesa is identified as the main source of illicit cigarettes seized in 2014, with the brand *Ibiza* comprising “10% of the domestic market (when considering both legal and illegal markets)” [translated by the authors] [99]. In 2014, it was reported that Tabesa produced six of the top selling contraband cigarette brands found in Mexico [100]. Given the above, journalist Mauri König and colleagues nicknamed Horacio Cartes, elected Paraguay's president in 2013, “The Tobacco Lord”, and called cigarette smuggling a “state business” in Paraguay [101].

Finally, our findings suggest that Tabesa’s business strategy, hitherto focused on the regional illicit tobacco trade, has begun to explore markets beyond Latin America. Comprehensive official data on Paraguayan companies’ exports of tobacco products remains unavailable, and Tabesa’s website does not list all of its export markets. However, in a 2011 media interview, Juan Carlos López Moreira (then CEO of Tabacos del Paraguay and Tabacos USA) disclosed, “We export to Switzerland, Mongolia, Romania, Bulgaria, the Canary Islands, Holland.” [translated by the authors] [86]. In an interview in 2012, Cartes mentions Switzerland, the US and Aruba as export markets, along with “some Arab countries” [translated by the authors] [22]. In 2014, Ortiz named Curaçao, in addition to the US and Aruba, among “other countries” [translated by the authors] [102].

Using these media interviews as a starting point, to search the UN Comtrade Database for officially reported cigarette exports by Paraguay, we found significant discrepancies between reported exports and imports of cigarettes, far greater than can be explained by the methodological challenges noted in the Methods section of this paper. For 10 of 11 territorial entities, official exports were greater than imports. Between 2001 and 2016, Paraguay reported exports of 23.6 million cigarettes to Switzerland, while Switzerland registered imports of only 3% of that amount – with reported imports of 766,076 cigarettes from Paraguay, a difference of almost 23 million cigarettes. During those same years, Paraguay registered exports of 111.4 million cigarettes to the Netherlands. However, there is no record by the Netherlands of any cigarette import from Paraguay. Similarly, Paraguay registered exports of 1.4 billion cigarettes to Curaçao, and 481.2 million cigarettes to the Netherlands Antilles, but neither report equivalent imports from Paraguay. The difference between official exports and imports during the same period amounted to 122.2 million cigarettes for Romania (where 68.9% of Paraguay’s reported exports were unaccounted for), 259.8 million cigarettes for Spain (67.3%), 49.2 million cigarettes for the UAE (64%), 3.1 billion cigarettes for Aruba (25.6%), and 138 million cigarettes for the US (6.1%). Aruba is a notable example given the country’s small size of just over 100, 000 inhabitants and low smoking prevalence rates of 16.2% for 25–64 year olds [103]. Despite this, Paraguay reported exports of 12.3 billion cigarettes between 2001 and 2016 (767.7m cigarettes on average per year). In 2001, 2002, 2013, and 2014, 2.6 million cigarettes were registered as exports from Paraguay to Aruba (including 2.1 million in 2013 alone), but there were no registered imports by Aruba. In Mongolia, an additional 9 million cigarettes were registered as imports. Finally, there is no official record of any Paraguayan exports of tobacco products to Bulgaria or any imports to Bulgaria from Paraguay between 2001 and 2016, despite Lopez Moreira listing the country as an export destination of Tabesa’s cigarettes in his 2011 media interview.

In total, Paraguay reported exports of 17.2 billion cigarettes to ten markets between 2001 and 2016, while these markets reported imports of only 11.5 billion cigarettes, leaving one-third of exports or 5.7 billion cigarettes unaccounted for. These discrepancies suggest significant diversion to the illicit market. Furthermore, available data is likely to be an underestimation. As noted above, evidence suggests large volumes of cigarettes produced by Tabesa are smuggled to countries where the company does not officially export to, primarily Brazil as well as Argentina, Colombia, and Mexico (Table 2).

**Table 2:** Cigarette trade between Paraguay and Tabesa’s named markets overseas (2001–2016). Source: UN Comtrade, using Iglesias et al. [48] conversion (1 kg = 956.4 cigarettes)

Trade partners	Cigarette exports reported by Paraguay	Cigarette imports reported by trade partner	Discrepancies (exports - imports)
Aruba	12,283,231,698	9,134,807,849	3,148,423,849
Curaçao	1,405,225,130	0	1,405,225,130
Netherlands Antilles	481,190,663	0	481,190,663
Spain	386,069,988	126,320,356	259,749,632
US	2,278,224,181	2,140,187,926	138,036,255
Romania	177,520,273	55,297,135	122,223,138
Netherlands	111,413,905	0	111,413,905
UAE	76,915,601	27,680,129	49,235,472
Switzerland	23,633,600	766,076	22,867,524
Bulgaria	0	0	0
Mongolia	15,072,864	24,031,463	-8,958,599

## Discussion

The above findings analyze the factors contributing to Tabesa’s rapid growth since the mid-1990s, into a major tobacco company in the region, and growing player globally. Leading TTCs had developed a substantial market for illicit tobacco products in Brazil and Argentina beginning in the 1960s. By the 1990s, their focus on low-priced brands, coupled with the Brazilian export tax of 1999, created a lucrative opportunity for Paraguayan companies to manufacture and supply their own brands to the illicit market. Tabesa was further enabled by a regulatory environment in Paraguay that was permissive and weakly governed. These findings of this paper support the conclusion by Joossens and Raw that, beyond tax differentials, the illicit trade is due to “other important factors [including] the ease and cost of operating in a country, industry participation, how well organized crime networks are, the likelihood of being caught, the punishment if caught, corruption levels” [8]. These factors appear to explain why a domestic tobacco company in Paraguay rose to outperform traditional TTCs in the sub-regional illicit market, and then aspire to pursue markets globally. Using independent data sources, we suggest Tabesa’s complicity in the illicit trade has extended from an initial focus on Latin America. Tabesa’s global expansion was achieved by creating new low-priced brands, improving quality, expanding manufacturing capacity, and then exporting its brands on a large scale through legal and illegal channels. Legal exports to the US, in addition have been used to enhance corporate reputation and fend off allegations of illegal activity.

There are four main implications for strengthening tobacco control, in Paraguay and globally, arising from these findings. First, the FCTC entered into force in Paraguay on 25 December 2006, although the country has yet to implement some of its key measures. Of particular relevance is Article 5.3, to protect tobacco control policies “from commercial and other vested interests of the tobacco industry in accordance with national law” [104]. WHO guidelines on Article 5.3 implementation urges that “government institutions and their bodies should not have any financial interest in the tobacco industry” [105]. On his first day in office, Cartes stated he was willing to sell Tabesa: “if someone’s paying, they can take my cigarette factory” [106]. In a 2016 interview, prompted by the publication of an article by the current authors on Tabesa’s alleged role in the illicit trade [20], CEO José Ortiz confirmed that Cartes remained a major shareholder [107]. Beyond the FCTC, this revelation has prompted questions under Article 237 of Paraguay’s Constitution which states: “The President of the Republic and the Vice President may not exercise public or private offices, remunerated or not, while in their functions. They may neither exercise commercial, industrial or any professional activity” [translated by the authors] [108]. At the time of writing, whether holding shares in a company should be interpreted as such is yet to be determined under Paraguayan domestic law. The findings of this paper suggest that the close relationship between a Paraguayan political leader and the tobacco industry is not conducive to a strong regulatory environment for tobacco control including effective action on the illicit tobacco trade. It is recognised here that tobacco control in Paraguay was relatively weak, compared to other FCTC States Parties, before Cartes’ election in 2013. Under Cartes, however, Tabesa officials became well-positioned to hinder stronger tobacco control, taxation, law enforcement, and other policy areas related to company interests via their direct working relationship with the president and his cabinet [109, 110]. Notably, FCTC Article 15 on the illicit trade in tobacco products has yet to be implemented in Paraguay. Authorities have not taken any significant steps to “[determine] the point of diversion and monitor, document and control the movement of tobacco products and their legal status”, “monitor and collect data on cross-border trade in tobacco products, including illicit trade”, “enact or strengthen legislation, with appropriate penalties and remedies, against illicit trade in tobacco products”, or “adopt measures as appropriate to enable the confiscation of proceeds derived from the illicit trade in tobacco products”, as the treaty stipulates.

Second, this paper highlights the importance for Paraguay and neighbouring countries to sign, ratify, and implement the WHO Protocol to Eliminate Illicit Trade in Tobacco Products. If the Protocol were fully implemented, tobacco company officials could no longer claim that, after selling to distributors, they are not responsible for the onward illegal sale of their products. Under the traceability system of the Protocol, detailed information on the whole supply chain is required, including “the name, invoice, order number and payment records of the first customer not affiliated to the manufacturer”, “the intended market of retail sale”, “any warehousing and shipping”, “the identity of any known subsequent purchaser”, and “the intended shipment route, the shipment date, shipment destination, point of departure and consignee” (Article 8.4.1). Article 10.1.b also stipulates that Parties shall take the necessary measures so that companies [supply] tobacco products or manufacturing equipment in amounts commensurate with the demand for such products within the intended market of retail sale or. This would prevent Tabesa and other companies from flooding small markets with cigarettes which are then diverted to illicit trade channels. Our methodology for estimating cigarette production finally shows the need to monitor and control key inputs in cigarette manufacturing, including tobacco leaf, cigarette paper, and acetate tow [7].

Third, the findings regarding Tabesa’s complicity in the illicit tobacco trade, as a core part of its business strategy, support the need for fuller understanding of the growing risk to global health from aspiring TTCs. The analytical framework of Lee and Eckhardt, within the context of tobacco industry globalization, and the methods and data sources used to estimate the scale of the ITTP from Paraguay, have proven useful for this purpose. As Lee and Eckhardt write, emerging TTCs will bring “fiercer competition for market share, especially in emerging markets”, likely leading to downward pressures on price, product innovation and intensified marketing [5]. This, in turn, will encourage increased consumption of products responsible for 7 million deaths annually by 2017 [111]. The findings also suggest links between aspiring TTCs, the illicit trade and organized crime [79–81]. As Joossens and Raw describe, organized crime is an important part of a global supply chain of harmful illicit substances including tobacco [8]. Further research to deepen understanding of the intertwined nature of the legal and illegal tobacco trade, the actors involved, and the transnational networks sustaining their activities, would benefit from integrating analytical frameworks from business studies and criminology.

Finally, this analysis of Paraguay points to the need for independent data and other resources to effectively implement the FCTC Protocol. BAT and PMI have commissioned several studies on the illicit tobacco trade in Latin America and Tabesa’s role in it [112, 113], and have framed the issue in the regional media to focus on Tabesa [114–116]. In turn, close relationship between the Cartes administration and tobacco industry between 2013-2018 cast doubt on Paraguyan government statements and figures on the illicit tobacco trade from Paraguay. As WHO cautions, “the involvement of organizations or individuals with commercial or vested interests in the tobacco industry in public health policies with respect to tobacco control is most likely to have a negative effect” [105].

## Conclusion

Tabesa represents an emerging threat to global health as a tobacco company expanding its markets worldwide, largely through the illegal trade of low-priced cheap brands. The continuing expansion of Tabesa is part of ongoing tobacco industry globalization which has seen a growing role for aspiring TTCs in some countries. While attention to the global business strategies of leading TTCs remains critical to global tobacco control, how non-TTCs may be working in cooperation and/or competition with TTCs requires fuller analysis. Since the 1960s, Paraguay has moved from a key hub for illicit trade of TTC brands within the subregion, to a major source of illicit tobacco products worldwide, led by the growth of Tabesa. Effective implementation of the FCTC and its Protocol will require fuller understanding of the dynamic and complex nature of the illicit tobacco trade worldwide, including improved data on the activities of such non-TTCs.

## Abbreviations

BAT: British American Tobacco
CIET: Centro de Investigación de la Epidemia de Tabaquismo - Tobacco Epidemic Research Center
DNP: Duty Not Paid
FARC: Revolutionary Armed Forces of Colombia (Fuerzas Armadas Revolucionarias de Colombia or FARC in Spanish)
FCTC: Framework Convention on Tobacco Control
FDI: Foreign Direct Investment
ICIJ: International Consortium of Investigative Journalists
KPMG: Klynveld Peat Marwick Goerdeler
Mercosur: Mercado Común del Sur (Spanish) or Mercado Comum do Sul (Portuguese) – Southern Common Market
MSA: Master Settlement Agreement
NIH: National Institutes of Health
PCC: First Capital Command (Primeiro Comando da Capital or PCC in Portuguese)
PMI: Philip Morris International
SA: Sociedad Anónima – Anonymous Company
TTCs: Transnational Tobacco Companies
TTID: Truth Tobacco Industry Documents
UAE: United Arab Emirates
UN Comtrade: United Nations Commodity Trade Statistics Database
UN: United Nations
USPTO: US Patent and Trademark Office
VGV: Valla Global Ventures
WHO: World Health Organization

## Footnotes

1 The Netherlands Antilles, whose islands included Curaçao, were part of the Netherlands until October 2010. The UN Comtrade website notes that data for the Netherlands does not include these islands. https://comtrade.un.org/ITSY2016VolI.pdf.

2 We chose the UAE because of Cartes’ reference to an ‘Arab country’ in a media interview. Euromonitor International notes that the UAE’s Jebel Ali Free Zone (JAF) is “increasingly central to the trade in cheap whites”, acting “as a production centre…[and] re-consignment hub for illicit white brands manufactured in Europe, Asia and Paraguay.” See Euromonitor International. Trends in illicit trade. *Tobacco Journal International*. 29 April 2015.

## References

[1] Lee K, Eckhardt J, Holden C (2016). Tobacco industry globalization and global health governance: towards an interdisciplinary research agenda. Palgrave Commun.

[2] Campaign for Tobacco Free Kids. The Global Cigarette Industry. 2016. https://www.tobaccofreekids.org/assets/global/pdfs/en/Global_Cigarette_Industry_pdf.pdf. Accessed 1 May 2018.

[3] Bilano V, Gilmour S, Moffiet T, Tursan d’Espaignet E, Stevens G, Commar A, Tuyl F, Hudson I, Shibuya K (2015). Global trends and projections for tobacco use, 1990–2025: an analysis of smoking indicators from the WHO Comprehensive information Systems for Tobacco Control. Lancet.

[4] Lee K, Eckhardt J (2017). The globalisation strategies of five Asian tobacco companies: an analytical framework. Global Public Health.

[5] Lee K, Eckhardt J (2017). The looming threat of Asian tobacco companies to global health. Lancet.

[6] Brookes N. Paraguay. BAT. 26 October 1994. https://www.industrydocumentslibrary.ucsf.edu/tobacco/docs/yzym0212. Accessed 30 July 2018.

[7] Iglesias R, Gomis B, Carrillo Botero N, Shepherd P, Lee K. From transit hub to major supplier of illicit cigarettes to Argentina and Brazil: The changing role of domestic production and transnational tobacco companies in Paraguay between 1960 and 2003. Glob Health. 2018;14:94. 10.1186/s12992-018-0413-2 PMC6245621 30454015

[8] Joossens L, Merriman D, Ross H, Raw M (2010). The impact of eliminating the global illicit cigarette trade. Addiction.

[9] WHO. Illicit trade in tobacco: A summary of the evidence and country responses. Presentation. 2012. http://www.who.int/tobacco/economics/illicittrade.pdf. Accessed 1 May 2018.

[10] LeGresley E, Lindblom EN (2002). Illegal Pathways to Illegal Profits: The big cigarette companies and international smuggling.

[11] Reuter P, Majmundar M (2015). Understanding the U.S. illicit tobacco market: characteristics, policy context, and lessons from international experiences.

[12] Bialous S (2015). The tobacco industry and the illicit trade in tobacco products.

[13] Holden C, Whyte D, Wiegratz J (2016). Transnational tobacco companies and the moral economy of cigarette smuggling. Neoliberalism and the economy of fraud.

[14] Collin J, LeGresley E, MacKenzie R, Lawrence S, Lee K (2004). Complicity in contraband: British American Tobacco and cigarette smuggling in Asia. Tob Control.

[15] MacKenzie R, Collin J, Sopharo C (2004). Sopheap Y. “Almost a role model of what we would like to do everywhere”: British American Tobacco in Cambodia. Tob Control.

[16] Lee K, Collin K (2006). “Key to the future”: British American Tobacco and cigarette smuggling in China. PLoS Med.

[17] LeGresley E, Lee K, Muggli ME, Patel P, Collin J, Hurt RD (2008). British American Tobacco and the “insidious impact of illicit trade” in cigarettes across Africa. Tob Control.

[18] Nakkash R, Lee K (2008). Smuggling as the “key to a combined market”: British American Tobacco in Lebanon. Tob Control.

[19] Gutierrez D. The smoking trail, cigarette smuggling in Paraguay. Harvard International Review. May 2016. http://hir.harvard.edu/article/?a=13337. Accessed 1 May 2018.

[20] Gomis B, Botero N. Sneaking a Smoke. Foreign Affairs. February 2016. https://www.foreignaffairs.com/articles/paraguay/2016-02-05/sneaking-smoke. Accessed 1 May 2018.

[21] Walker Guevara M, Rehnfelt M, Soares M. Smuggling made easy. Tobacco underground. ICIJ. 29 June 2009. https://www.icij.org/project/tobacco-underground/smuggling-made-easy. Accessed 1 May 2018.

[22] Cartes reconoce que sus cigarrillos llegan al mercado negro de Argentina y Brasil. ABC color. 6 March 2012. http://www.abc.com.py/nacionales/cartes-reconoce-que-suscigarrillos-llegan-al-mercado-negro-de-argentina-y-brasil-373906.html. Accessed 21 Mar 2018.

[23] Shepherd P, Newfarmer R (1985). Transnational corporations and the international cigarette industry. Profits, Progress and Poverty: case studies of International Industries in Latin America.

[24] Shepherd P (1983). “Soooold American!!!”: a study of the development of the foreign operations of the American cigarette industry. Ph.D. dissertation.

[25] Díaz Alejandro CF (1970). Essays on the economic history of the Argentine Republic.

[26] Abreu M, Carneiro Netto DD (1990). A Ordem do Progresso: cem anos de política econômica republicana, 1889–1989.

[27] Shepherd P, Teichova A, Levy-Leboyer M, Nussbaum H (1989). Transnational corporations and the denationalization of the Latin American cigarette industry. Historical studies in international corporate business.

[28] Palermo S.A. Empresa: De Tabacos del Paraguay S.A. a Palermo S.A. http://palermo.com.py/empresa.html. Accessed 21 March 2018.

[29] Subsecretaría de Tributación - Ministerio de Hacienda. Listado De Los 500 Principales Aportantes A La Set - Año 2015. 19 May 2016. http://www.set.gov.py/portal/PARAGUAY-SET/detail?folder-id=repository:collaboration:/sites/PARAGUAY-SET/categories/SET/Estadistica/ranking-de-mayores-aportantes-al-fisco&content-id=/repository/collaboration/sites/PARAGUAY-SET/documents/estadistica/ranking-de-contribuyentes-con-mayor-aporte-al-fisco/contribuyentes-con-mayores-aportes-ano-2015.pdf. Accessed 21 Mar 2018.

[30] According to XE.com. Accessed 24 Feb 2017.

[31] Euromonitor International. Passport database. Company Sshares. Cigarettes. Retail volume. 2017. http://go.euromonitor.com/passport.html. Accessed 18 Sept 2018.

[32] Euromonitor International. Tobacco in Uruguay. Country report. August 2016. http://www.euromonitor.com/tobacco-in-uruguay/report. Accessed 21 Mar 2018.

[33] Euromonitor International. Cigarettes in Bolivia. Country report. August 2016. http://www.euromonitor.com/cigarettes-in-bolivia/report. Accessed 21 Mar 2018.

[34] “TABESA currently accounts for 50% of total national production”. Grupo Cartes. Tabacalera del Este S.A. http://www.grupocartes.com.py/?portfolio=tabacalera-del-este-s-a [translated by the authors] Accessed 21 Mar 2018.

[35] “Market Share Año 2015: Tabacalera del Este S.A.: 53.9%”. Tabesa. Market share. http://www.tabesa.com.py/market-share.html. Accessed 21 June 2017.

[36] “Tabesa leads the market with a 53.5% share in 2013 with PMI in second position with 23.9% and BAT third with 20%.” ERC Group. World Cigarettes – Paraguay 2014. 21 November 2014. https://www.marketresearchstore.com/report/world-cigarettes-americas-2014-market-research-report-1476. Accessed 24 Feb 2017.

[37] “Tabesa is the leading domestic producer supplying over 50% of legitimate national production”. Canadean. Cigarettes in Paraguay. CG0295MR. September 2016. https://www.reportbuyer.com/product/4169141/cigarettes-in-paraguay.html Accessed 24 Feb 2017.

[38] MacKenzie R, Collin J, Lee K. The tobacco industry documents: an introductory handbook and resource guide for researchers. Centre on Global Health and change, London school of hygiene and tropical medicine, 2003. https://escholarship.org/uc/item/5c82b367. Accessed 8 Mar 2018.

[39] Anderson SJ, McCandless PM, Klausner K (2011). Tobacco documents research methodology. Tob Control.

[40] Gallagher AWA, Evans-Reeves KA, Hatchard JL, Gilmore AB. Tobacco industry data on illicit tobacco trade: a systematic review of existing assessments. Tob Control. (epub). 10.1136/tobaccocontrol-2018-054295. 10.1136/tobaccocontrol-2018-054295 PMC6580768 30135114

[41] UNODC (2009). World Drug Report 2009.

[42] Bunck JM, Fowler MR (2012). Bribes, bullets, and intimidation. Drug trafficking and the law in Central America.

[43] Gomis B. Demystifying ‘narcoterrorism’. Swansea: Global Drug Policy Observatory brief; 2015.

[44] Fooks G, Peeters S, Evans-Reeves K (2013). Illicit trade, tobacco industry-funded studies and policy influence in the EU and UK. Tob Control.

[45] Rowell A, Evans-Reeves K, Gilmore AB (2014). Tobacco industry manipulation of data on and press coverage of the illicit tobacco trade in the UK. Tob Control.

[46] Gomis, B. Beware of tobacco industry funding. Canadian International Council. 2017. https://thecic.org/beware-tobacco/. Accessed 18 Oct 2018.

[47] UN Comtrade. https://unstats.un.org/unsd/comtrade_announcement.htm. Accessed 21 Mar 2018.

[48] Iglesias R, Nicolau J. A economia do controle do tabaco nos países do Mercosul e associados: Brasil: PAHO; 2006. http://pesquisa.bvsalud.org/bvsms/resource/pt/mis-19172. Accessed 8 Mar 2018

[49] International Trade Centre. Consistency of Trade Statistics User Guide. Trade Competitiveness Map. http://tradecompetitivenessmap.intracen.org/Documents/TradeCompMap-Consistency%20of%20Trade%20Statistics-UserGuide-EN.pdf. Accessed 21 Mar 2018.

[50] World Bank. Imports, exports and Mirror data with UN COMTRADE. 2010. http://wits.worldbank.org/wits/wits/witshelp/Content/Data_Retrieval/T/Intro/B2.Imports_Exports_and_Mirror.htm. Accessed 21 Mar 2018.

[51] Merriman, D. Understand, Measure, and combat tobacco smuggling. World Bank economics of tobacco toolkit, Tool 7. 2003. http://siteresources.worldbank.org/INTPH/Resources/7Smuggling.pdf. Accessed 1 May 2018.

[52] Canadian Institutes of Health Research (2014). Tricouncil Policy Statement, Ethical Conduct for Research Involving Humans.

[53] National Institutes of Health. Guiding principles for ethical research. Washington, DC; 2016. https://www.nih.gov/health-information/nih-clinical-research-trials-you/guiding-principles-ethical-research. Accessed 21 Mar 2018

[54] Shafey O, Dolwick S, Guindon E (2003). Editors. Paraguay. In tobacco control country profiles, second edition.

[55] PAHO (2016). Report on tobacco control for the region of the Americas.

[56] WHO (2017). WHO report on the global tobacco epidemic, 2017.

[57] Euromonitor International. Passport database. Accessed on 21 June 2017. http://go.euromonitor.com/passport.html

[58] Drope J, Schluger N (2018). Paraguay, Country Fact Sheet. Tobacco Atlas.

[59] Central Intelligence Agency. The World Factbook. https://www.cia.gov/library/publications/the-world-factbook/geos/pa.html. Accessed 21 Mar 2018.

[60] Jerberg C (2009). Brazil and tobacco use: a tough nut to crack. Bull World Health Organ.

[61] Grupo Cartes. Tabacos USA. http://www.grupocartes.com.py/?portfolio=tabacos-usa-inc. Accessed 21 Mar 2018.

[62] Tabacos USA. http://www.tabacosusa.com. Accessed 21 Mar 2018.

[63] Holden C, Lee K, Fooks G, Wander N (2010). The impact of regional trade integration on firm organisation and strategy: British American Tobacco in the andean pact. Bus Polit.

[64] Sáenz de Miera-Juárez B, Iglesias R (2010). Impuestos para el control del tabaquismo: las experiencias de Brasil y México. Salud Publica Mex.

[65] Iglesias R, Pinto M, da Costa e Silva VL, Godinho J (2007). Tobacco control in Brazil. Discussion paper.

[66] Matamos a los gringos con calidad y por eso lloran. ABC color. 29 June 2009. http://www.abc.com.py/edicion-impresa/economia/matamos-a-los-gringos-con-calidad-y-por-eso-lloran-1186531.html. Accessed 21 Mar 2018.

[67] Connelly M (1995). The transhipment problem: smuggling and welfare in Paraguay. World Dev.

[68] U.S. Department of State (2015). Paraguay. International narcotics control strategy report.

[69] Neumann V. Towing the line: controlling key inputs to disrupt illegal tobacco. MacDonald-Laurier institute. Anti-Contraband Tobacco Working Group Workshop, 25 November 2014. http://www.macdonaldlaurier.ca/files/pdf/Appendix%20F%20-%20Neumann%20Presentation.pdf. Accessed 21 Mar 2018.

[70] Interview with former law enforcement officer, 15 Apr 2015.

[71] de Miera-Juarez B, Iglesias R (2010). Taxation and tobacco control: the cases of Brazil and Mexico. Salud Publica Mex.

[72] Tabesa. http://www.tabesa.com.py/en/. Accessed 21 June 2017.

[73] Palermo. http://www.palermo.com.py. Accessed 21 Mar 2018.

[74] Ramos A. Illegal trade in tobacco in MERCOSUR countries. Working Paper: CIET Uruguay; 2009. http://fctc.wpengine.com/wp-content/uploads/2009/06/INB3_report_illegal_trade_in_MERCOSUR.pdf; later published in: Ramos, A. Illegal trade in tobacco in MERCOSUR countries. Trends in Organized Crime 2009; 12(3/4): 267

[75] Holden C (2017). Graduated sovereignty and global governance gaps: special economic zones and the illicit trade in tobacco products. Polit Geogr.

[76] Grupo Cartes. Valla Global Ventures. http://www.grupocartes.com.py/?portfolio=valla-global-ventures-ltd. Accessed 21 Mar 2018.

[77] Import Genius. Sample shipment record for Valla global ventures Ltda. 2009. https://www.importgenius.com/paraguay/buyers/valla-global-ventures-ltda. Accessed 21 Mar 2018.

[78] Import Genius. Sample shipment record for Valla global ventures Ltda. 2010. https://www.importgenius.com/suppliers/valla-global-ventures-ltd. Accessed 21 Mar 2018.

[79] Paraguay President Profits from Mexico Contraband Cigarettes. Insight crime. 9 December 2014. http://www.insightcrime.org/news-briefs/paraguay-president-profits-from-mexico-contraband-cigarettes. Accessed 21 Mar 2018.

[80] Colombia Criminals Use Paraguay Contraband Cigarettes to Launder Money. Insight crime. 24 March 2014. http://www.insightcrime.org/news-briefs/colombia-criminals-use-paraguay-contraband-cigarettes-to-launder-drug-money. Accessed 21 Mar 2018.

[81] Cigarette Seizures Highlight Paraguay Contraband Trade. Insight crime. 29 May 2014. http://www.insightcrime.org/news-briefs/brazil-cigarette-seizures-highlight-paraguay-contraband-trade. Accessed 21 Mar 2018.

[82] BAT. Briefing Document in Relation to the CPI Report of 31 January 2000 Dealing with Latin America. 11 February 2000. https://www.industrydocumentslibrary.ucsf.edu/tobacco/docs/fmyj0210. Accessed 21 Mar 2018.

[83] Ortiz: Tabesa nada tiene que ver con contraband. ABC Color. 13 June 2016. http://www.abc.com.py/nacionales/ortiz-tabesa-nada-tiene-que-ver-con-contrabando-1489148.html. Accessed 21 Mar 2018.

[84] USPTO. Trademark Status & Document Retrieval (TSDR). http://tsdr.uspto.gov/#caseNumber=2516146&caseSearchType=US_APPLICATION&caseType=DEFAULT&searchType=statusSearch. Accessed 16 Mar 2017.

[85] Grupo Cartes. Tabacalera del Este S.A. http://www.grupocartes.com.py/?portfolio=tabacalera-del-este-s-a. Accessed 21 Mar 2018.

[86] ABC Color. Directivos de tabacalera de Cartes desconocen investigación de EE.UU, revelada por WikiLeaks. 24 October 2011. http://www.abc.com.py/nacionales/directivos-de-tabacalera-de-cartes-desconocen-investigacion-de-eeuu-revelada-por-wikileaks-325454.html. Accessed 21 Mar 2018.

[87] Panjiva. https://panjiva.com. Accessed 21 Mar 2018.

[88] BuzzFile. Tabacos USA, Inc. Business Description (Online). http://www.buzzfile.com/business/Tabacos-USA-Inc-610-438-2005. Accessed 19 Feb 2017.

[89] Johnson, S. Letter to Commissioner of Trademarks, USPTO. Re: Declaration of use of mark in commerce – Section 8; Application for renewal of registration – Section 9 Palermo Brand Cigarettes. 3 May 2012.

[90] ZA-90-0007/YAZ1K Martinetti, Julio et al/operation heart of stone case coordination meeting. 5 January 2010. https://wikileaks.org/plusd/cables/10BUENOSAIRES5_a.html. Accessed 21 Mar 2018.

[91] Managing Expections of New SEPRELAD Director. 7 August 2007. https://wikileaks.org/plusd/cables/07ASUNCION714_a.html. Accessed 21 Mar 2018.

[92] Horacio Cartes: Millionaire. Criminal. Business titan. Homophobe. The next president of Paraguay? Independent. 19 April 2013. http://www.independent.co.uk/news/world/americas/horacio-cartes-millionaire-criminal-business-titan-homophobe-the-next-president-of-paraguay-8580851.html. Accessed 21 Mar 2018.

[93] Departamento Estadistica, Adminstración Sistems SOFIA, Dirección Nacional de Aduanas. Accessed 21 June 2017.

[94] Kentucky. Palermo. http://www.palermo.com.py/kentucky.html. Accessed 21 Mar 2018.

[95] BITQ Consulting Services Ltd, 2015-2016 data.

[96] ABC Color. Incautan cigarillos paraguayos. 30 December 2016. http://www.abc.com.py/nacionales/incautan-cigarrillos-paraguayos-1551947.html

[97] Gazeta do Povo. O patrão de tabaco. Império das Cinzas. 22 March 2014. http://www.gazetadopovo.com.br/vida-e-cidadania/especiais/imperio-das-cinzas/o-patrao-do-tabaco-20c56zz6kbdqqr7boh6hdv9la. Accessed 21 Mar 2018.

[98] Iglesias R, Szklo A, Souza M, de Almeida L (2017). Estimating the size of illicit tobacco consumption in Brazil: findings from the global adult tobacco survey. Tob Control.

[99] El Espectador. En la ruta del Tabaco. 18 November 2014. http://www.elespectador.com/noticias/infografia/ruta-del-tabaco-articulo-528217 Accessed 21 Mar 2018.

[100] Milenio. Enriquece contraband a president paraguayo. 8 December 2014. http://www.milenio.com/politica/Enriquece_contrabando_presidente_paraguayo-contrabando_de_tabaco-cigarrillos_ilegales_0_423557650.html. Accessed 21 Mar 2018.

[101] Gazeta do Povo. Império Das Cinzas. Especiais. March/April 2014. http://www.gazetadopovo.com.br/vida-e-cidadania/especiais/imperio-das-cinzas/. Accessed 21 Mar 2018.

[102] Portal Politico TV. Tabacalera paraguaya de presidente Cartes niega contrabando a México. 9 December 2014. http://www.portalpolitico.tv/internacionales/tabacalera-paraguaya-de-presidente-cartes-niega-contrabando-a-mexico. Accessed 21 Mar 2018.

[103] Central Bureau of Statistics. STEPS ARUBA 2006: Risky Living. http://cbs.aw/wp/wp-content/uploads/2013/02/STEPS_ARUBA_2006_Risky_Living.pdf. Accessed 20 Mar 2018.

[104] WHO. WHO FCTC. 2003. http://www.who.int/fctc/text_download/en/. Accessed 21 Mar 2018.

[105] WHO. Guidelines for implementation of article 5.3. 2008. http://www.who.int/fctc/guidelines/article_5_3.pdf. Accessed 21 Mar 2018.

[106] Associated Press. Paraguay’s tobacco-growing president says he’ll sell off his cigarette factory. 16 August 2013. https://article.wn.com/view/2013/08/16/Paraguay_s_tobaccogrowing_president_says_he_ll_sell_off_his_/ Accessed 21 Mar 2018.

[107] ABC Color. Cartes sigue siendo accionista de Tabesa. 9 February 2016. http://www.abc.com.py/nacionales/contradicciones-sobre-contrabando-de-tabaco-1451731.html. Accessed 21 Mar 2018.

[108] Constitución de la República de Paraguay, 1992. http://www.hacienda.gov.py/normativa/Constitución%20de%20la%20República%20de%20Paraguay.pdf Accessed 21 Mar 2018.

[109] Los asesores oficiales de Cartes pisan fuerte en áreas claves de la economía. Ultima Hora. 2 March 2014. http://www.ultimahora.com/los-asesores-oficiales-cartes-pisan-fuerte-areas-claves-la-economia-n771411.html. Accessed 21 Mar 2018.

[110] Cartes’ choice of aides sparks new scandal. Buenos Aires Herald. 26 February 2014.

[111] WHO. Tobacco. Fact sheet. May 2017. http://www.who.int/mediacentre/factsheets/fs339/en/. Accessed 21 March 2018.

[112] KMPG. Project Frost. London: KPMG; 2015. https://cigarroilicito.weebly.com/uploads/1/0/9/4/109477265/project_frost_final_spanish_26_06_2015.pdf. Accessed 18 Sept 2018.

[113] KPMG. Project EOS: A study of illicit cigarette consumption in Latin America and Canada. London: KMPG; 2017. https://static1.squarespace.com/static/58e3aecb5016e199be776da1/t/5980c11a03596e503f425620/1501610267901/EOS+2016+-+Canada+%28002%29.pdf. Accessed 18 Sept 2018.

[114] Paraguay.com. Gigantes del Tabaco financian investigación contra Cartes. 25 March 2014. http://www.paraguay.com/judiciales-policiales/gigantes-del-tabaco-financian-investigacion-contra-cartes-105094?ep=true. Accessed 4 Apr 2018.

[115] La Nacion. La economía paraguaya se hace humo. 22 June 2003. https://www.lanacion.com.ar/505611-la-economia-paraguaya-se-hace-humo. Accessed 4 Apr 2018.

[116] La Nacion. Proviene del contraband el 20% de los cigarrillos. 21 November 2005. https://www.lanacion.com.ar/758205-proviene-del-contrabando-el-20-de-los-cigarrillos

